# Generation of reactive oxygen species mediates butein-induced apoptosis in neuroblastoma cells

**DOI:** 10.3892/or.2012.1632

**Published:** 2012-01-12

**Authors:** YA-HUI CHEN, CHI-WEI YEH, HUI-CHEN LO, SHIH-LI SU, YOU-CHENG HSEU, LI-SUNG HSU

**Affiliations:** 1 Institute of Biochemistry and Biotechnology, Chung Shan Medical University, Taichung; 2 Department of Medical Education and Research, Changhua Christian Hospital, Changhua; 3 Department of Nutritional Science, Fu Jen Catholic University, New Taipei City; 4 Department of Medical Education and Research, Changhua Christian Hospital, Changhua; 5 Division of Endocrinology and Metabolism, Department of Internal Medicine, Changhua Christian Hospital, Changhua; 6 Department of Cosmeceutics, College of Pharmacy, China Medical University, Taichung; 7 Clinical Laboratory, Chung Shan Medical University Hospital, Taichung, Taiwan, R.O.C

**Keywords:** apoptosis, butein, neuroblastoma, reactive oxygen species

## Abstract

Flavonoids exhibit chemopreventive and chemotherapeutic effects. Butein, a bioactive flavonoid isolated from numerous native plants, has been shown to induce apoptosis in human cancer cells. In the current study, the molecular mechanisms of butein action on cell proliferation and apoptosis of neuroblastoma cells were evaluated. Treatment with butein decreased the viability of Neuro-2A neuroblastoma cells in a dose- and time-dependent manner. The dose-dependent nature of butein-induced apoptosis was characterized by an increase in the sub-G1 phase population. Treatment with butein significantly increased intracellular reactive oxygen species (ROS)levels and reduced the Bcl-2/Bax ratio, triggering the cleavage of pro-caspase 3 and poly-(ADP-ribose) polymerase (PARP). Pre-treatment with the antioxidant agent, N-acetyl cysteine (NAC), blocks butein-induced ROS generation and cell death. NAC also recovers butein-induced apoptosis-related protein alteration. In conclusion, butein-triggered neuroblastoma cells undergo apoptosis via generation of ROS, alteration of the Bcl-2/Bax ratio, and cleavage of pro-caspase 3 and PARP. Our results suggest that butein may serve as a potential therapeutic agent for the treatment of neuroblastoma.

## Introduction

Epidemiological reports show that the consumption of vegetables and fruits rich in polyphenols can reduce the incidence of various diseases, such as cardiovascular disease (1), allergic inflammation (2), Alzheimer’s disease (3), and cancer (4). Butein (3,4,2′,4′-tetrahydroxychalcone), a bioactive polyphenol isolated from the stem bark of cashews and *Rhus verniciflua* Stokes, has been used as a food additive and traditional herb medicine (5). Butein exhibits antioxidant and anti-inflammatory activities (6,7). It also exerts antitumor activities against a wide range of cancers, including colon cancer (8), osteosarcoma (9), lymphoma (5), melanoma (10), and breast cancer (11). Treatment with butein modulates the Bcl-2/Bax ratio, increases pro-caspase 3 activity, and subsequently triggers apoptosis in cancer cells (9,12). In addition, co-treatment of butein with TNF-related apoptosis inducing ligand (TRAIL) enhances death receptor 5 (DR5) expression and elevated caspase 3 activity, which, in turn, cause TRAIL-resistant leukemia and hepatoma cell lines to undergo apoptosis (13,14). Very recently, butein was found to inhibit the migration of breast and pancreatic cancer via abrogated nuclear factor κB (NFκB) activity and downregulated chemokine receptor CXCR4 expression and function (15).

Neuroblastoma, a highly prevalent solid tumor in children, accounts for 15% of all pediatric cancer-related deaths worldwide (16). Due to its easy metastasis and drug resistance, the mortality of neuroblastoma patients is very high. Therefore, determination of novel drugs that induce apoptosis in neuroblastoma cells without causing toxicity to normal cells is of utmost importance in the treatment of neuroblastoma. In the current study, we show for the first time that butein markedly triggers apoptosis in neuroblastoma cells through the generation of reactive oxygen species (ROS), alteration of Bcl-2 and Bax expression, and elevation of pro-caspase 3 activity. Pre-treatment with antioxidant agents reverse butein-induced effects. In general, butein may be a potential chemotherapeutic agent for the effective treatment of neuroblastoma.

## Materials and methods

### Materials

Butein, 3-(4,5-dimethylthiazol-2-yl)-2,5-diphenyltetrazolium bromide (MTT), NAC, and propidium iodide were purchased from the Sigma Chemical Company (St. Louis, MO, USA). Antibodies against pro-caspase 3 and PARP were purchased from Cell Signaling Technology (Beverly, MA, USA). Antibodies against Bcl-2 and Bax were obtained from Santa Cruz Biotechnology (Santa Cruz, CA, USA). Anti- β-actin and HRP-conjugated secondary antibodies were obtained from Sigma Chemical Company.

### Cell culture

Mouse neuroblastoma Neuro-2A cells were maintained in modified Eagle’s medium (Gibco; Gaithersburg, MD, USA) supplemented with 10% fetal bovine serum (FBS), 100 U/ml penicillin and 100 g/ml streptomycin (Gibco) at 37°C in a humidified 5% CO_2_ incubator.

### MTT assay

Cells were seeded in 24-well plates at a density of 4×10^4^/ml and treated with the indicated concentrations of butein for 24 or 48 h. After removal of the supernatant, the cells were incubated with a medium containing 5.0 g/l MTT and incubated at 37°C for an additional 3 h. The formazan was dissolved in 1 ml isopropanol and the absorbance was measured at 563 nm. The experiments were performed in duplicate and repeated at least 3 times.

### Cell cycle distribution and apoptosis analysis

Cells exposed to different concentrations of butein for 24 h were collected and fixed with ice-cold ethanol overnight at −20°C. Subsequently, cells were stained with 50 μg/ml propidium iodide (PI) in the dark for 15 min. The cell cycle distribution was analyzed on a BD Biosciences FACScan system using the CellQuest™ Pro software. The proportion of hypodiploid cells (sub-G1 phase) was also analyzed.

### Reactive oxygen species (ROS) analysis

Cells were pretreated using 2 mM NAC for 1 h, followed by treatment with an indicated concentration of butein for an additional 24 h. The cells were then loaded with a 5 μM fluorescent probe 2′,7′-dichlorodihydrofluorescein diacetate (H2DCFDA) at 37°C for 1 h. The fluorescent intensity was analyzed using Molecular Devices Flexstation 3.

### Western blot analysis

Cells were treated with the indicated concentration of butein for 24 h. Cells were rinsed with ice-cold phosphate buffered saline (PBS) and lysed in buffer containing 150 mM NaCl, 1% Triton X-100, 5 mM EDTA, 5 mM phenylmethylsulfonyl fluoride, 1% aprotinin, 1 g/ml leupeptin, and 500 μM Na_3_VO_4_. The protein concentration was detected using Bradford protein assay kit (Bio-Rad Laboratories, Hercules, CA, USA). Twenty micrograms of protein were separated by 10% polyacrylamide gel and transferred into a nitrocellulose membrane. The membrane was blocked by PBS containing 0.5% non-fat milk for 1 h at room temperature. After washing with PBS containing 0.1% Tween-20 (PBST), the membrane was probed with the indicated antibodies at 4°C overnight, washed with PBST, and subsequently incubated with horseradish peroxidase-conjugated goat anti-mouse IgG antibody (Santa Cruz Biotechnology, Inc., Santa Cruz, CA; 1:5,000 dilution) at room temperature for 1 h. The bands were detected by enhanced chemiluminescence kit (Perkin-Elmer Life Science, Boston, MA, USA). β-actin expression was used as the loading control.

### Statistical analysis

Data reported are the mean ± SD deviation of 3 independent experiments and evaluated by Student t-tests. Significant differences were established at P<0.05.

## Results

### Effects of butein on the viability and apoptosis of neuroblastoma cells

**Figure 1:**
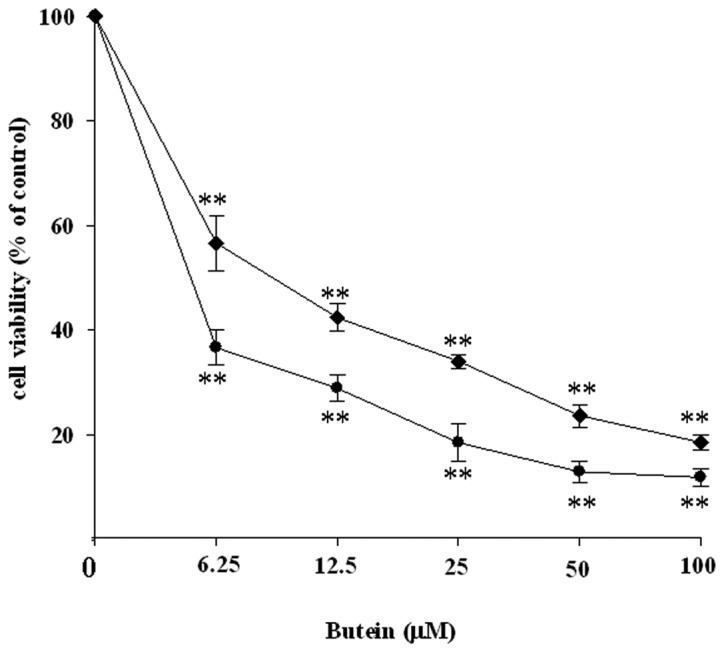
Butein decreases cell viability of mouse neuroblastoma Neuro-2A cell. Neuro-2A cells were treated with different concentrations of butein for 24 (^♦^) and 48 h (^•^). The cell viability was determined by the MTT assay. The viability of the vehicle group (0.1% DMSO) was set to 100%. The data represent the mean ± SD from at least 3 independent experiments.

To determine the effects of butein on the cell viability of neuroblastoma, Neuro-2A cells were treated with various concentrations of butein for 24 and 48 h. Cell viability was measured by the MTT assay. After treatment with 6.25, 12.5, 25, 50, and 100 μM butein for 24 h, cell viabilities were determined to be around 56.65, 42.41, 34.08, 23.68 and 18.58%, respectively, compared with the vehicle control. Cell viabilities were 36.70, 28.91, 18.54, 12.98 and 11.91% compared with the vehicle control when the cells were exposed to 6.25, 12.5, 25, 50 and 100 μM butein for 48 h. Collectively, treatment with butein resulted in a dose- and time-dependent inhibition of cell viability in Neuro-2A cells (Fig. 1).

**Figure 2:**
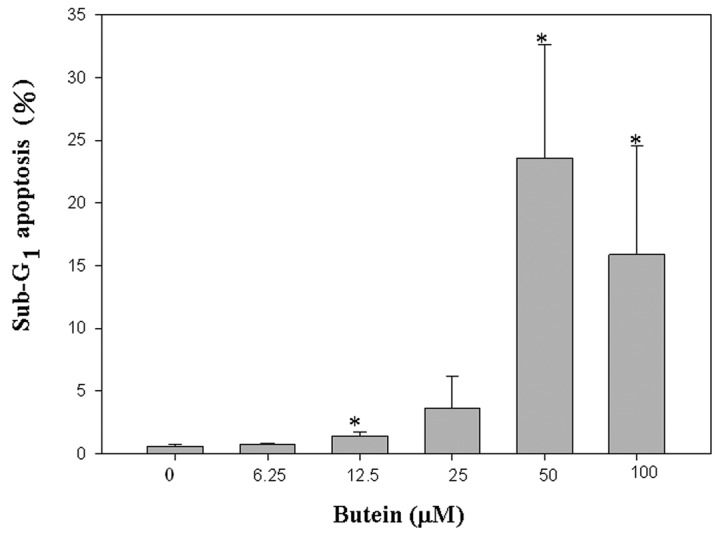
Butein induces apoptosis in Neuro-2A cells. Cells treated with the indicated concentrations of butein were collected and subjected to flow cytometry analysis with propidium iodide staining. The apoptotic cell (sub-G1 population) content was counted and the quantitative data are represented mean ± SD from 3 independent experiences. ^*^P<0.05.

To further examine the inhibitory effects of butein on cell viability, cell cycle progression was conducted by flow cytometry analysis. Increased sub-G1 phase percentages were found in proportion to the butein treatments (0.58, 0.71, 1.45, 3.63, 23.60 and 15.89% in the presence of 0, 6.25, 12.5, 25, 50, and 100 μM butein). Accordingly, treatment with butein diminished cell viability mainly by triggering apoptosis (Fig. 2).

### Effects of butein on apoptosis-related protein expression

**Figure 3:**
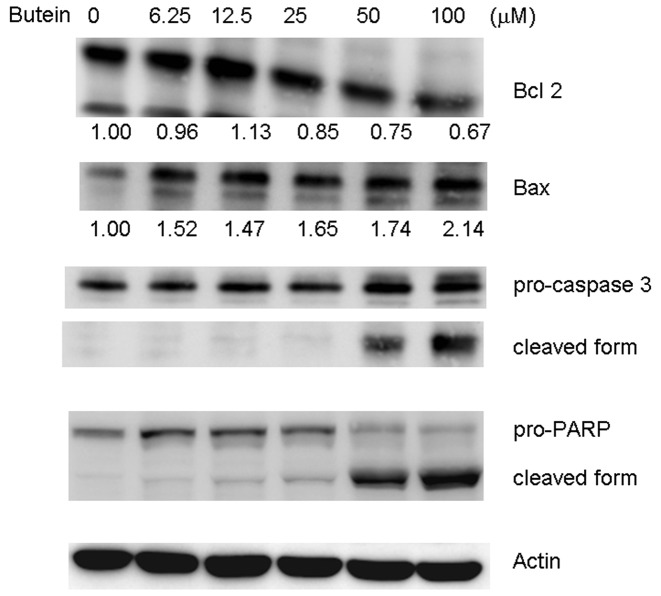
Effects of butein on apoptosis-related protein expression. Neuro-2A cells were treated with the indicated concentrations of butein for 24 h. Cell lysates were harvested and subjected to Western blot analysis using antibodies against Bcl-2, Bax, pro-caspase 3, and PARP. The data represent one of the 3 independent experiments. β-actin was used as a loading control. The intensities of Bcl-2 and Bax were quantified and compared to the vehicle-treated group.

Bcl-2 family proteins are involved in apoptosis (17,18). Decreased Bcl-2/Bax ratios may trigger the release of cytochrome C from the mitochondria and activation of pro-caspase 3 (18). To elucidate the role of Bcl-2 family proteins in butein-induced apoptosis, the expressions of Bcl-2, Bax, pro-caspase 3, and PARP were measured in cell lysates derived from butein-treated cells. In the presence of 25, 50, and 100 μM butein, Bcl-2 expression decreased by 15, 25 and 30%, respectively, compared with the vehicle control. Bax expression increased by 1.52, 1.47, 1.65, 1.74 and 2.14-fold compared with the control (Fig. 3). Significantly reduced pro-forms and increased cleavage forms of caspase 3 and PARP were also observed in the butein-treated groups (Fig. 3).

### Effect of antioxidant agents on butein-induced changes

**Figure 4:**
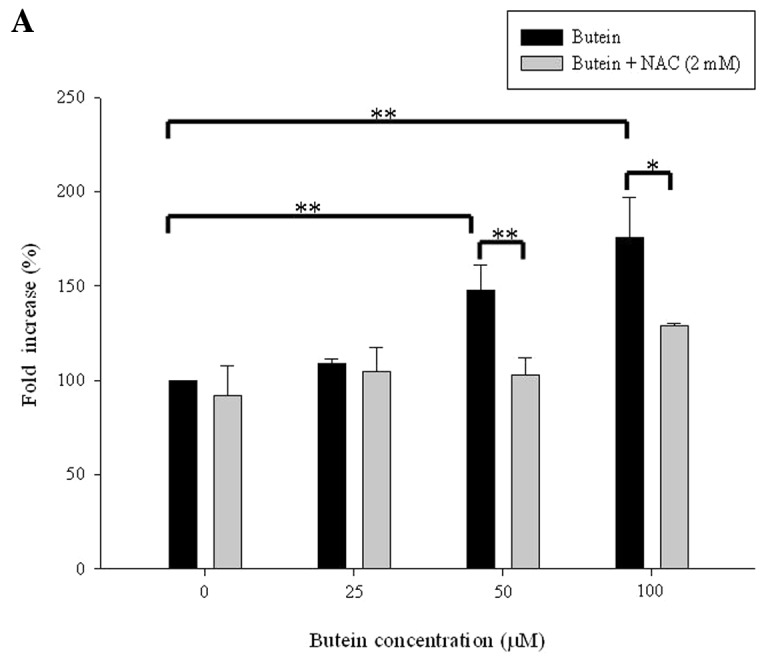

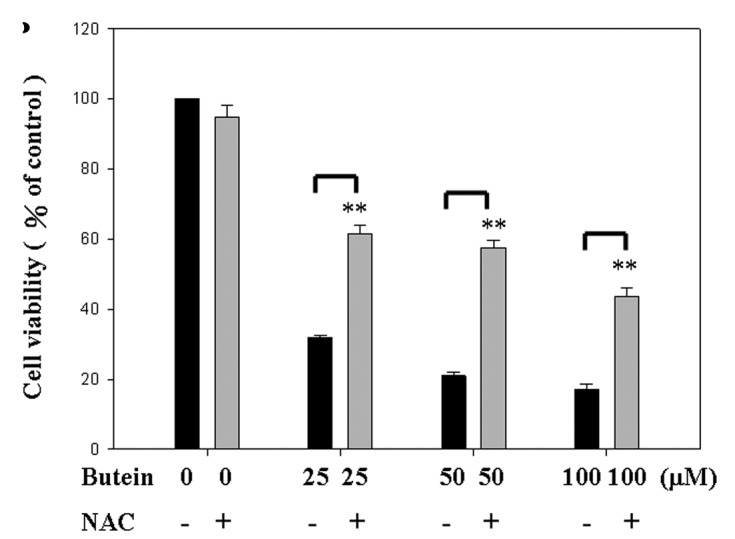
Effects of NAC on butein-induced (A) ROS generation and (B) cell viability. Neuro-2A cells were pre-treated with 2 mM NAC, followed by co-treatment with the indicated concentration of butein for an additional 24 h. ROS generation was measured by the DCFDA staining method. Cell viability was determined by the MTT assay. ^*^P<0.05; ^**^P<0.001.

Recent studies demonstrated that generation of ROS may function as a mediator in flavonoid-induced apoptosis in cancer cells (19,20). To determine whether or not butein induces oxidative stress, ROS generation by butein was determined using the DCFDA staining method. As expected, treatment with 25, 50 and 100 μM butein significantly induced ROS production. ROS generation was diminished by pretreatment with the antioxidant agent NAC (Fig. 4A).

To determine whether or not elevated ROS levels mediate the cytotoxic effects of butein, the effects of antioxidant agents on cell viability was examined. Cells were pretreated with 2 mM NAC and then treated with 25, 50 and 100 μM butein for an additional 24 h. As shown in Fig. 4B, butein-induced cell death was significantly prevented by NAC. In the presence of NAC, cell viability recovered to about 61.56, 57.53 and 43.50% in the presence of 25, 50, and 100 μM butein compared to the vehicle-treated group, respectively.

**Figure 5:**
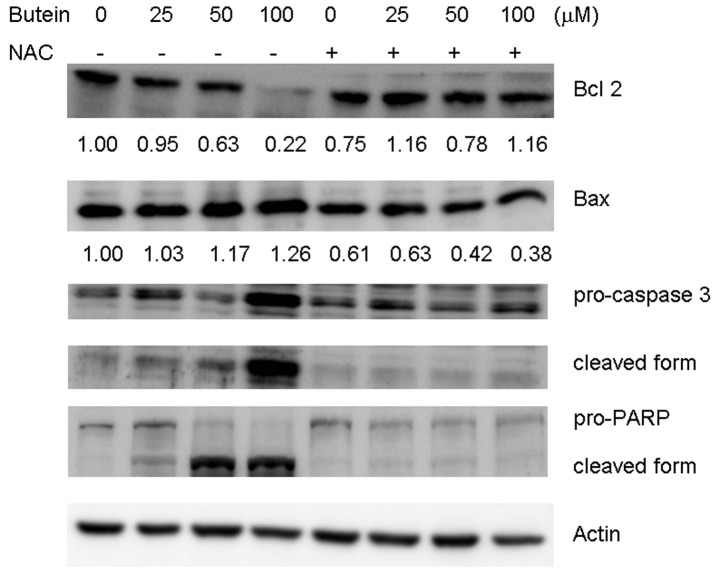
NAC reverses butein-induced apoptotic protein expression. Neuro-2A cells were pretreated with 2 mM NAC, followed by co-treated with the indicated concentrations of butein for additional 24 h. Cell lysates were harvested and subjected to Western blot analysis using antibodies against Bcl-2, Bax, pro-caspase 3, and PARP. The data represent one of the 3 independent experiments. β-actin was used as a loading control. The intensities of Bcl-2 and Bax were quantified and compared to the vehicle treated group.

To determine whether or not NAC reverses apoptosis-related protein expression, cell lysates derived from combined treatments with NAC and butein were subjected to Western blot analysis. NAC significantly recovered the expression of Bcl-2 and attenuated the expression of Bax expression (Fig. 5). Cleavage form of caspase 3 and PARP was also prevented by NAC. Taken together, our results show that ROS plays a critical role in butein-induced apoptosis.

## Discussion

Extensive reports have revealed the inverse relationship between the consumption of vegetables and fruits and several chronic diseases, such as allergic inflammation, hypertension, and cancer (21,22). Flavonoids, one of the major bioactive compounds in vegetables and fruits, exhibit anti-atherosclerosis (23), anti-inflammation (4), and antitumor effects (4). Butein, an ingredient isolated from native plants, inhibits the proliferation of several human cancers, such as melanoma (10), leukemia (12), and osteosarcoma (9). In this presentation, the molecular mechanisms of butein on the apoptosis of neuroblastoma cells were delineated for the first time. Butein inhibited the cell viability of neuroblastoma cells in a dose- and time-dependent manner. Treatment with butein induced apoptosis, as evidenced by an increased percentage of the sub-G1 population. In addition, butein decreased the Bcl-2/Bax ratio, and enhanced the cleavage of caspase 3 and PARP. Pretreatment with antioxidant agents such as NAC clearly attenuated butein-induced effects.

The balance of expression of pro-apoptotic and anti-apoptotic members of the Bcl-2 family proteins plays a critical role in apoptosis (24). An increased pro-apoptotic/anti-apoptotic protein ratio altered the membrane potential of the mitochondria, released apoptogenic factors to the cytoplasma, activated the caspase cascade, and eventually caused apoptosis (18,24). Crude acetone extracts of *R. verniciflua* Stokes, which is rich in fustin and fisetin, and butein-triggered osteosarcoma cells undergo apoptosis via the activation of Bax and the down-regulation of Bcl-2 expression (9). Similarly, butein increased Bax expression and decreased Bcl-2 expression in HL60 leukemia cells (12). In accordance with previous reports, our findings also showed that butein upregulated Bax expression and attenuated Bcl-2 expression in neuroblastoma cells. Moreover, activation of caspase 3 and cleavage of PARP, 2 hallmarks of apoptosis, were also observed in butein-treated Neuro-2A cells. Collectively, our data suggest that butein-induced apoptosis may mediate by increase ROS level and change the pro-apoptotic/anti-apoptotic protein ratio.

Alternation of intracellular ROS production may modulate several physiological functions (25). Elevated ROS can bind to lipids, proteins, or DNA to produce oxidative stress and eventually cause cell death (25). Flavonoid-induced apoptosis in cancer cells via elevation of intracellular ROS levels has been shown in several reports (19,20), although previous reports have also shown that butein induces ROS generation, modulates JNK, ATM, and Chk activity, and causes G2/M arrest in hepatoma cells (26). The consequence of butein-induced ROS production in cell death remains unclear. In the current presentation, butein enhanced ROS levels up to 1.5-fold and caused cell death. Blockage of ROS generation by an antioxidant agent, NAC, significantly increased cell viability in the presence of butein. Cotreatment with NAC recovered the anti-apoptotic protein Bcl-2 and decreased pro-apoptotic Bax levels, which, in turn, attenuated pro-caspase 3 activity. ROS appears to play a pivotal role in butein-induced apoptosis in neuroblastoma cells.

In summary, to the best of our knowledge, this is the first report to show that butein inhibits cell viability in neuroblastoma cells. Butein triggers apoptosis, as evidenced by an increase in the sub-G1 population. Butein also elevated ROS levels, altered Bcl-2/Bax ratios, induced pro-caspase 3 and PARP cleavage, and consequently caused cells to undergo apoptosis. Pre-treatment with NAC obviously abrogated the butein-induced effects. Collectively, our findings suggest that butein may serve as a chemotherapeutic agent for the future treatment of neuroblastoma.
